# The impact of electronic prescribing systems on healthcare professionals’ working practices in the hospital setting: a systematic review and narrative synthesis

**DOI:** 10.1186/s12913-019-4554-7

**Published:** 2019-10-22

**Authors:** Soomal Mohsin-Shaikh, Dominic Furniss, Ann Blandford, Monsey McLeod, Tiantian Ma, Maedeh Y. Beykloo, Bryony Dean Franklin

**Affiliations:** 1 UCL School of Pharmacy, Research Department of Pharmacy Practice and Policy, Entrance A, Tavistock Square, London, WC1H 9HR UK; 2 UCL Interaction Centre, University College London, London, UK; 3 Centre for Medication Safety and Service Quality, Imperial College Healthcare NHS Trust, London, UK

**Keywords:** Electronic prescribing, Electronic prescribing and medication administration systems, Working practices, Healthcare professionals, Inpatient

## Abstract

**Background:**

The aim of this systematic review was to synthesise peer-reviewed literature assessing the impact of electronic prescribing (eP) systems on the working practices of healthcare professionals (HCPs) in the inpatient setting and identify implications for practice and research.

**Methods:**

We searched PubMed, Medline, Embase, Cochrane and the Cumulative Index to Nursing Allied Health Literature databases for studies published from inception to November 2018. We included controlled, uncontrolled, observational and descriptive studies that explored the effect of eP on HCPs’ working practices in an inpatient setting. Data on setting, eP system and impact on working practices were extracted. Methodological quality was assessed using the Mixed Methods Appraisal Tool. Emergent themes were identified and subjected to narrative synthesis. The protocol was registered with PROSPERO (registration CRD42017075804).

**Results:**

Searches identified 1301 titles and abstracts after duplicate removal. 171 papers underwent full-text review. A total of 25 studies met the inclusion criteria, from nine different countries. Nineteen were of commercial eP systems. There were a range of study designs; most (*n* = 14) adopted quantitative methods such as cross-sectional surveys, ten adopted qualitative approaches and a further one used mixed methods. Fourteen of the 25 studies were deemed to be of high quality. Four key themes were identified: communication, time taken to complete tasks, clinical workflow, and workarounds. Within each theme, study findings differed as to whether the effects of eP on HCPs’ working practices were positive or negative.

**Conclusion:**

There is a lack of consensus within the literature on the impact of eP on HCPs’ working practices. Future research should explore the strategies resulting in a positive impact on HCPs’ working practices and learn from those that have not been successful.

## Background

Electronic prescribing (eP) and electronic prescribing and medication administration (ePMA) systems are increasingly being implemented in clinical settings in an attempt to reduce medication-related risks and enhance patient safety [1]. In some countries, the term computerised provider order entry (CPOE) is used instead in the hospital setting, where the scope of CPOE may also include other types of medical orders such as laboratory tests and radiology [1]. While there are many potential safety benefits, studies also suggest that adoption of new hospital health information technology introduces sociotechnical challenges that can limit these benefits [1, 2], often because such systems affect healthcare professionals’ (HCPs’) work and the way they perform their roles. Previous publications have highlighted successful implementation and use of electronic prescribing in primary care [3, 4]. A systematic review investigated the impact of hospital CPOE systems on inpatient clinical workflow, but included literature on all types of medical order and only up to June 2007 [5]. The focus of the present review is hospital eP, whether standalone or part of a wider ePMA and/or CPOE system. To the best of our knowledge, there have been no previous systematic reviews specifically exploring the effects of eP on the working practices of HCPs. For the purposes of this review, ‘working practices’ were considered to refer to HCPs conducting clinical work, diagnostics, monitoring, and/or interacting and communicating with other HCPs. Our aim was to synthesise peer-reviewed literature assessing the impact of eP systems on the working practices of HCPs in the inpatient setting and identify implications for practice and research.

## Methods

Searches were carried out in The Cochrane Library, PubMed, Medline, Embase, and the Cumulative Index to Nursing Allied and Health Literature databases across all publication dates up to 19 November 2018. The reference lists of included studies were also searched and an expert in the field was consulted to identify further relevant papers. The search strategy, constructed with the support of specialist librarians, included combinations of keywords and controlled vocabulary. The full search strategy for all five databases can be found in Additional file 1: Table S1.

The entire set of titles and abstracts was screened by a reviewer (SMS) and any duplicates removed. Following this, all titles, index terms, and abstracts (if available) were checked and each paper was classified as either “potentially relevant” or “not relevant” based on the inclusion and exclusion criteria in Table 1. A second independent reviewer (MM) reviewed a random 10% sample of the titles and abstracts. The full articles were then retrieved for all potentially relevant papers. A third reviewer (TM) independently screened a random 20% sample of the full papers. Any full text papers on which reviewers SMS and TM disagreed were reviewed by a fourth reviewer (BDF) and resolved by consensus. Inter-reviewer agreement for each stage was assessed using Cohen’s kappa [6].

**Table 1:** Inclusion and exclusion criteria

Criteria	Inclusion	Exclusion
Time period	All up until 19 November 2018	
Publication language	English	Any other publication language
Setting	Studies that were conducted in one or more hospital settings – general hospitals, specialist hospitals, teaching hospitals or any other type of hospital	Studies based in a primary care or outpatient setting: e.g. GP practices, ambulatory clinics, residential or nursing homes
	Any inpatient group – including adult and paediatric patients, medical, surgical and critical care patients	
Study design	Any study design, including controlled, uncontrolled (such as uncontrolled before-and-after studies), observational (including cohort and case-controlled studies), descriptive (such as surveys) or qualitative designs	Viewpoints, editorials, conference/meeting abstracts, expert opinions and grey literature. Systematic or similar reviews (e.g. narrative, scoping and realist reviews) were excluded but their references were reviewed to identify relevant studies
Study participants	Studies focusing on doctors, pharmacists and/or nurses working with hospital inpatients. If there were a mix of any other healthcare professionals (HCPs) within a study, the study was only included if the data among the HCP groups could be distinguished	Studies that focused on other healthcare professionals e.g. physiotherapists, dieticians, occupational therapists unless the data could be extracted for the included healthcare professionals
Intervention	Studies that focused on the impact of electronic prescribing (eP) systems on the working practices of healthcare professionals; these could include standalone eP systems or electronic prescribing and medication administration systems	Studies that focused on the impact of paper-based systems for prescribing without any comparison with eP systems
	The hospital could have a previously implemented eP system, or an eP system implemented during the course of the study	Studies that focused only on the introduction or impact of barcoded medication administration/clinical decision support/alerts/mobile health technology
	Papers related to the introduction or impact of barcode medication administration/clinical decision support/alerts/mobile health technology but with the main focus being eP	Studies of a standalone discharge prescription system or specialist chemotherapy eP system

### Inclusion and exclusion criteria

Inclusion and exclusion criteria are documented in Table 1.

### Quality assessment

The Mixed Methods Appraisal Tool (MMAT) [7] was used to assess studies’ methodological quality. This tool was selected as it can be used with quantitative, qualitative and mixed-methods studies. A random sample of 50% of the included papers underwent quality assessment by two authors (SMS and MB) independently and any disagreements on study quality were resolved by discussion. Quality assessment was then conducted by SMS for the remaining studies. Based on this appraisal tool, studies were awarded a score of unclassified, 25, 50, 75% or 100%, with scores of 75–100% considered high quality. Studies were not excluded based on quality but quality scores were presented descriptively.

### Data extraction and analysis

A data extraction template was created to allow standardisation of the data collated from each included study and piloted before first use. For each article included, the reviewer (SMS) extracted information regarding the study aim, setting, the type/brand of eP and the outcomes measures used. A narrative synthesis was used to describe the findings; the anticipated heterogeneity of study designs, outcome measures and systems studied precluded any form of meta-analysis.

The Economic and Social Research Council (ESRC) guidance was used to conduct the narrative synthesis [8] and the Preferred Reporting Items for Systematic Reviews and Meta-Analyses (PRISMA) statement used to guide reporting [9]. The protocol was registered with PROSPERO international prospective register of systematic reviews (registration CRD42017075804).

## Results

**Fig. 1:**
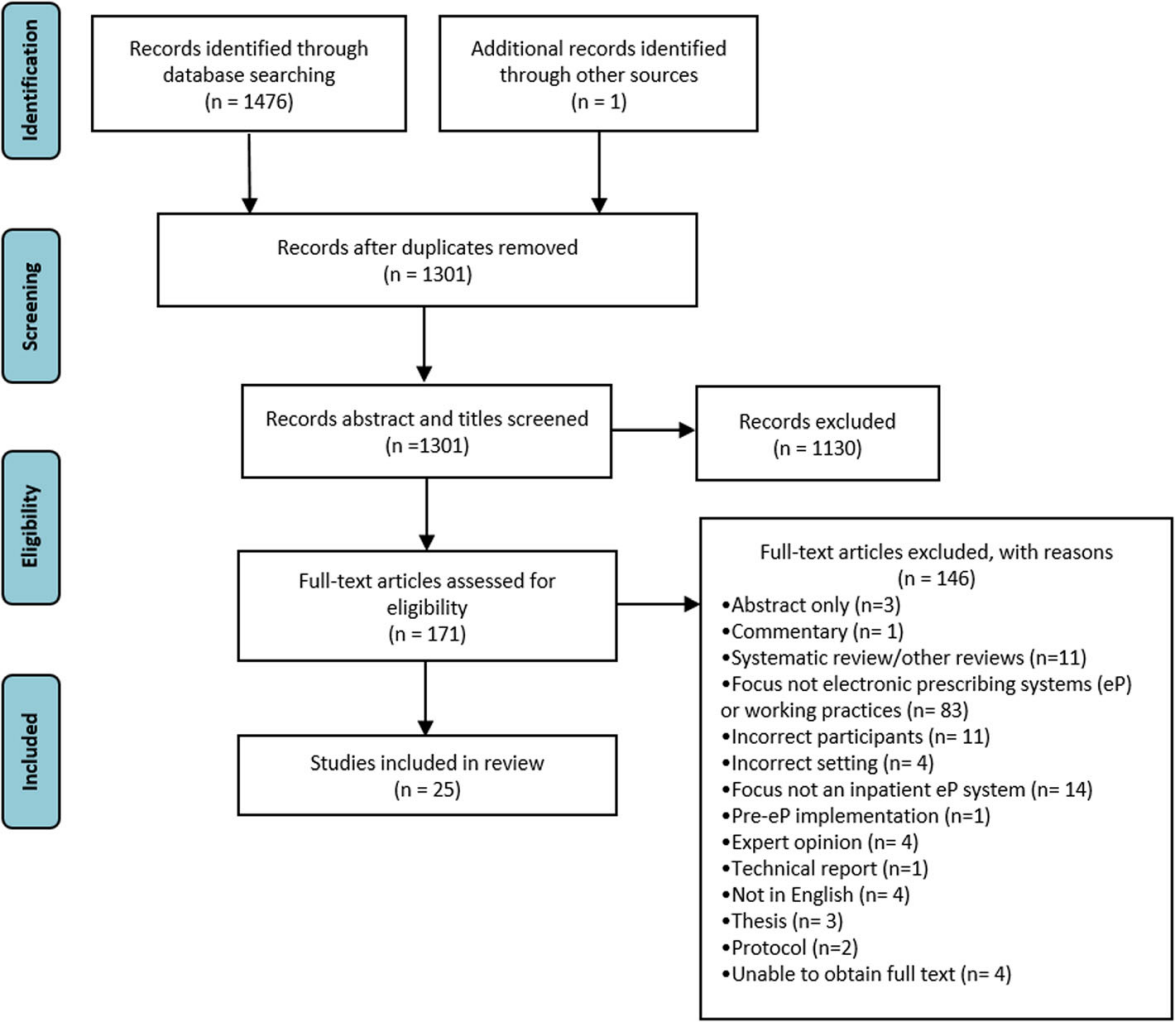
PRISMA diagram of flow of studies, PRISMA chart summarising the screening process

The search identified 1477 articles. Following deduplication and exclusion of four non-English papers, 1301 articles underwent title and abstract screening. The titles and abstracts for 130 of these were screened by the second researcher (MM). Based on title and abstract review, 171 articles underwent full-text screening; of these, 34 were also screened by TM. The inter-reviewer agreement was deemed almost perfect for the title and abstract screening (Cohen’s kappa = 0.948) and moderate for the full-text screening (Cohen’s kappa = 0.637, 9). Six papers were reviewed by BDF and their inclusion or exclusion agreed by consensus. The screening process is summarised in Fig. 1. Twenty-five studies met the inclusion criteria. The full data extraction table can be found in Additional file 2: Table S2. The MMAT quality assessment was completed by two reviewers for 13 studies, with strong inter-reviewer agreement (Cohen’s kappa = 0.816). Once all the studies were assessed, ten were rated 100%, four as 75%, ten as 50%, and one as 25% (see Additional file 3: Table S3). Fourteen of 25 studies were therefore deemed high quality (75–100% score).

Of the 25 studies, seven were from the UK [10–16], four from the US [17–20], four from the Netherlands [21–24], three from France [25–27], two from Australia [28, 29], two from Saudi Arabia [30, 31] and one each from Denmark [32], Spain [33] and Iran [34]. Nineteen studied commercial systems, one a home-grown system [25], one both a commercial and a home grown [15] system and for four studies it was not possible to establish the system type [11, 30, 31, 34]. Nineteen studies referred to their electronic systems as CPOE, and among these, three specified that the system was for prescribing medication only [22–24]. The remaining six papers studied ePMA systems, although in two cases these were referred to as eP [12, 14]. Sixteen papers studied CPOE systems with electronic medication administration and three studied CPOE without electronic medication administration. The six papers that studied ePMA systems included one exploring a mix of ePMA systems and one that studied a standalone eP system. The included studies used a range of data collection methods and study designs, mainly cross-sectional. Most applied quantitative methods (*n* = 14) such as surveys, ten applied qualitative approaches including focus groups, interviews and observations, and one used mixed-methods [26]. Across the 25 studies, nurses were included in 18, doctors in 17 and pharmacists in 9 studies. Four key themes were derived from the studies’ findings: communication, time taken to complete tasks, clinical workflow, and workarounds.

### Communication

Twelve papers highlighted the impact of eP systems on HCPs’ communication among professions. Two reported a positive impact on HCPs [17, 20], two reported no significant difference [27, 29], three reported a negative impact [22, 23, 26] and five reported a preference for verbal communication over electronic [11, 15, 21, 25, 31]. Two specifically reported a positive impact on doctor-nurse communication since introduction of eP [17, 20]. In one of these, interviewed doctors perceived that communication with colleagues and nurses improved through better documentation [17]. Similarly, in the second study, nurses reported adequate communication with doctors when using eP [20]. Furthermore, in this study and another qualitative study it was found that communicating orders electronically risked miscommunication between HCPs as there were no bedside systems to enter medication orders [22, 23]. The doctor therefore had to rely on their memory or write a brief note on paper to remind them to prescribe medication later [22, 23]. In another study, it was reported that eP systems benefited the doctor-pharmacy and nurse-pharmacy workflows but hindered doctor-nurse workflows as the unidirectional nature of medical dominance in the ordering phase caused nurses difficulties in their workflow [23].

Two studies revealed that both medical and nursing staff preferred verbal communication rather than communication through an eP system [21, 31]; this view was further supported in interviews conducted with doctors and pharmacists [15]. Nurses reported that they always supplemented eP communication with a phone call to confirm medication orders, which they perceived to add to their workload [21, 31]. Three studies focused on the impact of eP on pharmacists and their communication with doctors [11, 15, 25]. It was found that pharmacists’ interventions were well accepted by doctors when communicated both electronically and orally [25], but with a significantly higher acceptance rate for those communicated orally. This study also suggested that pharmacists preferred oral communication in situations requiring a rapid modification to medication [25]. These two studies also indicated that there was an increase in communication between doctor and pharmacist as eP introduced a new ‘technical’ expert role for pharmacists [11, 15], suggested to have evolved due to suboptimal doctors’ training [15].

Two studies reported no significant impact of eP systems on HCPs’ communication [27, 29]. One identified common rounds, briefings and opportunistic exchanges as opportunities for medical and nursing staff to exchange patient-related information, and then compared the impact of these on communication between an eP site and a paper-based site; no statistically significant difference in cooperative activities was identified [27]. Similarly, a controlled before-and-after time and motion study found that an electronic system was not associated with any significant change in the proportion of time medical and nursing staff spent in professional communication with each other [29].

### Time taken to complete tasks

Six papers focused on the impact of eP on time taken while completing particular medication-related tasks [13, 16, 18, 19, 28, 29]; the majority adopted uncontrolled before-and-after study designs (*n* = 4), one a controlled before-and-after design [29] and one was a longitudinal qualitative study [28]. Of the former, one focused on the impact of eP on nurses’ medication-related activities [16]. This study suggested that eP did not significantly affect the length of time spent on a medication administration round but altered the distribution of tasks with a doubling of the time spent on documentation [16]. The duration of the medication round appeared to decrease post-eP but data collection ceased before the reason for this change could be fully explored [16]. In another study in which HCPs were interviewed at four time points, doctors and nurses perceived that prescribing and medication administration took longer post-eP compared to paper medication charts. Six months post-eP, participants perceived that they had become more efficient in using the system but the time taken for medication administration had not returned to pre-eP durations as the process now included additional steps such as double signing for each dose administered [28].

One quantitative study explored the impact of a closed-loop eP system on staff time [13]. Nurses’ medication rounds took less time post-implementation but more time was required for medication-related tasks outside the medication round. Prescribing and pharmacists’ reviews took more time post-implementation. However, it was highlighted that since all medication charts were electronic, they were always accessible, so there was an increase in the number of charts available for pharmacists’ review, which could have contributed to the increase in time taken [13]. Conversely, another study found that time taken for a pharmacist to verify a medication order reduced compared to hand written orders [18]. This was because the eP system allowed some steps in the ordering process to be eliminated, contributing to time saved during the pharmacists’ review [18, 19]. Another study found that the time taken to communicate orders from the prescriber to the pharmacy and the time taken to dispense and administer medication to the patient improved [19]. In contrast, in a controlled study, the proportion of time taken for medical and nursing staff to complete medication-related tasks did not change relative to control wards [29].

### Impact on clinical workflow

Three papers concluded that nurses perceived the introduction of eP to positively impact their workflow [30, 33, 34]. However, in one of these, it was reported that nurses who believed they received substandard training for eP were less satisfied with their workflow than those who perceived their training to be fair or good [30]. In another study, nurses rated their post-eP workload as good or very good in comparison to doctors who rated theirs as fair or poor [33].

Three papers suggested a negative impact on nursing workflow following implementation of eP [10, 24, 32]. For example, in a qualitative study, nurses reported being hesitant to adopt the system at the start, feared letting go of familiar aspects of their job and expressed resistance to computers becoming a more substantial part of their role [10]. In a questionnaire study, responses of nurses switching from two different paper-based processes to eP reported that they would prefer to continue using the eP system, although the study also suggested that nurses believed that the new system did not support their work processes [24]. Another study also reported that eP did not support a ‘collaborative working environment’, as doctors and nurses were less likely to negotiate and discuss patient treatments together [32].

Doctors saw advantages in having the ability to enter orders within and outside the hospital, allowing easy access to legible patient information, but perceived that entering electronic orders took more time compared to the paper-based system [12, 28]. These perceptions support previous research that highlighted a longer duration for medications to be prescribed electronically [13, 33]. Doctors also expressed their frustrations by describing a new eP system as being time-consuming, as it impacted their perceptions of the system’s suitability and usability [28]. The notion of becoming over-dependent on the technology was suggested, but doctors perceived that having access to information improved clinical decision making [33]. However, the extra steps needed to obtain the information from the system were seen as a burden and an increase in workload [17, 28, 33]. Doctors had more negative responses towards the eP system compared to nurses and pharmacists [12].

Two papers presented pharmacists’ perception of the impact of eP systems on their workflow [11, 14]. In a small UK study, hospital pharmacists all highlighted that more clinical screening was being completed away from the ward and in one hospital the role of pharmacy technicians had changed to become more ward-based before the roll out of eP to support maintaining medication stock and dispensing items for the ward [14]. Since the pharmacists were relieved of conducting these tasks post-eP, they had more clinical input on the wards by attending ward rounds [14]. Five of seven hospital pharmacists interviewed believed the amount of time pharmacists spent on the ward had not changed and four reported that pharmacists visited all the patients daily whether they had a wireless or fixed device system [14]. This contradicts the findings from another UK study that suggested pharmacists conducted their work away from the patient due to the lack of available computers in patient areas following the introduction of an electronic system [11]. As eP systems offer the flexibility to complete remote screening, pharmacists in both studies were concerned about reduced patient contact and denying patients opportunities to ask questions [11, 14]. Furthermore, during focus groups, pharmacists who had been using an eP system for 8 months reported that reduced patient contact had resulted in poorer relationships with patients [11]. Pharmacists at three hospitals reported their pharmacy workload had increased while their pharmacy workforce remained the same [14]. In most cases, only one extra staff member (pharmacist, technician or system manager) was recruited to help implement and support the system [14].

### Workarounds

Two papers explored the introduction of workarounds in the context of eP [22, 28]. A number of workarounds were identified at each stage of the medication use process [22]. At the point of prescribing it was highlighted that often the computer terminal was not near the patient, thus, the review and prescribing of medication took place away from the patient and was reliant on the prescriber’s memory [22]. An example of a nursing workaround introduced following eP is nurses administering medication without an electronic prescription if the doctor was busy and not able to prescribe the medication at the patient’s bedside [22]. In this situation, the nurse would start to administer the medication based on the doctor’s verbal or paper-based order and either handwrite the order onto the medication record card (instead of affixing a label) or call the doctor to remind them to prescribe the medication [22]. In a paper-based environment, a handwritten order would satisfy the prescription requirements, but with the electronic system used in this study, in which nurses administered against paper records, additional steps were required to produce a valid prescription such as an electronic order and to print a prescription label for nurses to administer against. In a separate qualitative study, it was found that 6 months after eP implementation, workarounds were adopted to overcome limitations of slow computers. Nurses no longer took computers to the bedside and some nurses viewed this workaround to be less safe, as medication details and patient identification were no longer being checked immediately prior to medications being administered [28].

## Discussion

Our review suggests that the ‘devil is in the detail’; not only in the methods and measures used for the different eP studies, but also in how positive and negative outcomes may be affected by the nuances of the context and the implementations of technologies. Similar to the broader systematic review of CPOE conducted in 2009 [5], we found benefits to include legibility, remote access and reduced times for certain tasks. We also found that some processes were more time-consuming and restricted opportunities for team-wide discussion [5]. However, our review went beyond aspects of clinical workflow to also include studies detailing the impact of ePMA systems on HCPs’ communication, time taken to complete tasks, and workarounds. These new themes identified suggest that future research should focus on the impact of eP systems on different HCPs’ working practices but also on how the eP system can support different HCPs working together. Our findings also support those of a previous review of the barriers and facilitators to implementing eP systems in primary care [3]. They found that eP system users report benefits in saving time and improving efficiency [3]. As in to our review, challenges were also identified; including overdependence on technology and negative impact on workflow [3]. Our review suggested that users of eP and ePMA reported that the system often did not support their work processes and did not support a ‘collaborative working environment’ [24, 32]. In our review we also found that pharmacists were the least represented HCP group, included in only nine studies, suggesting them to be an under-researched profession. It could be argued that this reflects the fact that there are fewer pharmacists in the hospital setting compared to doctors and nurses. However, pharmacists play a key role in the medication use process in most hospital inpatient settings and are significant users of eP systems; thus, future research should explore the impact of eP on their working practices.

### Implications for practice

There is a lack of consensus within the literature on the impact of eP systems on HCPs’ working practices. eP systems have removed the need for certain medication-related tasks such as searching for paper medication charts [16]; conversely such systems have introduced other time-consuming tasks such as login procedures that can delay ordering and medication dose adjustments [32]. The literature implies that information is now accessible to all HCPs which has been considered both advantageous [28] and a burden [13]. There was a reported increase in workload for all three HCP groups discussed in this review [13–15, 23, 33] which could in turn put pressure on the workforce. Hospitals may therefore need to monitor their workload in relation to the available workforce and redistribute work among health professions. Workforce managers and senior HCPs should identify and take steps to address time-intensive tasks locally in order to maximise the benefits and minimise the shortcomings of eP. Managers should encourage staff working in hospitals to continue oral communication as studies have found that tasks are more likely to be acted on if communicated orally compared to electronic communication [25].

### Implications for research

This review has identified a number of gaps in the research as all four themes identified from the review require further exploration to draw more definitive conclusions. This review also reveals that relatively little research has been conducted on how pharmacists are affected by eP. There needs to be further research into understanding the impact of eP on their working practices. Importantly, we identified variability among studies and settings, which made it difficult to draw firm conclusions. Researchers should examine the differences among contexts, study designs and implementation strategies to facilitate future research and shed light on why there is such heterogeneity in study findings.

### Strengths and limitations

There are a number of strengths to this review. Unlike previous reviews, we focused on eP rather than CPOE to achieve a more focused review. The facets and keywords were generated by a rigorous process and with the support of previous literature and specialist librarians. To improve transparency and reduce risk of bias, a second researcher checked a sample of the papers at each stage of the review. Fourteen of the twenty-five studies were deemed of high quality (75–100%) and only one of very low quality (25%).

Despite the aforementioned strengths, there were also a number of limitations. We excluded studies not published in English, published only as abstracts and those that could not be retrieved, although each of these were small in number. The retrieved papers focused on different aspects of working practices for different HCPs, which made comparing findings difficult. It is also important to acknowledge international variation in the type, method and purpose of working practices relating to medication and therefore eP may be expected to have different effects in different contexts.

## Conclusion

HCPs continue to face practical challenges working with new and different technology and it is important to draw upon their positive and negative experiences with eP and ePMA systems to work towards refining healthcare systems. Researchers should further unpick why such heterogeneity exists among different studies. Little information regarding the usual practices and implementation strategies was provided in the included papers; it was therefore difficult to differentiate the impact of different contexts, settings and study designs. Researchers should further dissect the strategies put in place in certain settings that may have led to a positive impact on HCPs’ working practices and learn from those that have not been successful. HCPs and other stakeholders may be able to learn from settings where these systems have been beneficial to reduce any negative impact on the workforce.

## Supplementary information

**Additional file 1: Table S1.** Search strategy.

**Additional file 2: Table S2.** Data extraction table.

**Additional file 3: Table S3.** Quality assessment - The Mixed Methods Appraisal Tool (MMAT) version 2018.

## Data Availability

Data sharing is not applicable to this article as no datasets were generated or analysed during the current study.

## Abbreviations

CPOE: Computerised physician/provider order entry
eP: Electronic prescribing
ePMA: Electronic prescribing and medication administration
ESRC: Economic and Social Research Council
HCP(s): Healthcare professional(s)
PRISMA: Preferred Reporting Items for Systematic Reviews and Meta-Analyses
UK: United Kingdom
