# Supplementary material for: The impact of electronic prescribing systems on healthcare professionals’ working practices in the hospital setting: a systematic review and narrative synthesis
Source: BMC Health Serv Res. 2019 Oct 22;19:742. doi: 10.1186/s12913-019-4554-7 (PMC6806498; doi:10.1186/s12913-019-4554-7)
Supplement: Supplementary file 1 — Additional file 1: Table S1. Search strategy. [file 12913_2019_4554_MOESM1_ESM.docx]

| Cochrane (accessed on 19/11/18) | | | | |
| --- | --- | --- | --- | --- |
| MeSH term | | Keywords | | Truncations |
| Facet 1 | Electronic prescribing | N/A | | N/A |
|  | Medical order entry system | CPOE (Computerised provider/physician order entry) | | N/A |
| **Number of records identified: 123** | | | | |
| Medline (accessed on 19/11/18) | | | | |
| MeSH term | | Keywords | | Truncations |
| Facet 1 | Electronic prescribing | - Electronic prescribing - CPOE - Medical order entry system - Medication alert system - Computerised physician order entry - Computerised provider order entry | | e*prescri* or electronic prescri* or CPOE or medical order entry system* or medication alert system* or computeri*ed physician order entr* or computeri*ed provider order entr* |
|  | Medical order entry system |  |  |  |
| Facet 2 | Healthcare professionals | - Healthcare professional - Health personnel - Pharmacist - Doctor - Clinician - Physician - Hospital medical staff - Nurse - Hospital nursing staff - Registered nurse | | healthcare professional* or health care professional* or healthcare personnel or health personnel or pharmacist* or doctor* or clinician* or physician* or hospital medical staff or nurs* or hospital nursing staff or registered nurse* |
|  | Physician |  |  |  |
|  | Pharmacist |  |  |  |
|  | Nurse |  |  |  |
| Facet 3 | Inpatient | - Inpatient - Hospitalised patient - Hospital patient | | inpatient* or hospitali*ed patient* or hospital patient* |
| Facet 4 | Workflow | - Working practice - Workaround - Workflow - Practice pattern - Communication - Staff time | | work* practice* or workaround* or work flow* or workflow* or practice pattern* or communica* or staff time |
|  | Communication, Hospital communication, Interdisciplinary communication |  |  |  |
| **Number of records identified: 154** | | | | |
| **EMBASE (accessed on 19/11/18)** | | | | |
| MeSH term | | Keywords | | Truncations |
| Facet 1 | Electronic prescribing | - Electronic prescribing - CPOE - Medical order entry system - Medication alert system - Computerised physician order entry - Computerised provider order entry | | e*prescri* or electronic prescri* or CPOE or medical order entry system* or medication alert system* or computeri*ed physician order entr* or computeri*ed provider order entr* |
|  | Computerised provider order entry |  |  |  |
| Facet 2 | Health care personnel | - Healthcare professional - Health personnel - Pharmacist - Doctor - Clinician - Physician - Hospital medical staff - Nurse - Hospital nursing staff - Registered nurse | | healthcare professional* or health care professional* or healthcare personnel or health personnel or pharmacist* or doctor* or clinician* or physician* or hospital medical staff or nurs* or hospital nursing staff or registered nurse* |
|  | Physician |  |  |  |
|  | Pharmacist |  |  |  |
|  | Nurse, Staff nurse, Registered nurse |  |  |  |
| Facet 3 | Hospital patient | - Inpatient - Hospitalised patient - Hospital patient | | inpatient* or hospitali*ed patient* or hospital patient* |
| Facet 4 | Workflow | - Working practice - Workaround - Workflow - Practice pattern - Communication - Staff time | | work* practice* or workaround* or work flow* or workflow* or practice pattern* or communica* or staff time |
|  | Communication, Interpersonal communication |  |  |  |
| **Number of records identified: 177** | | | | |
| **CINAHL (accessed on 19/11/18)** | | | | |
| MeSH term | | Keywords | | Truncations |
| Facet 1 | Electronic order entry | - Electronic prescribing - CPOE - Medical order entry system - Medication alert system - Computerised physician order entry - Computerised provider order entry | | e*prescri* or electronic prescri* or CPOE or medical order entry system* or medication alert system* or computeri*ed physician order entr* or computeri*ed provider order entr* |
| Facet 2 | Health personnel | - Healthcare professional - Health personnel - Doctor - Clinician - Hospital medical staff - Nurse | | healthcare professional* or "health care professional*" or "healthcare personnel" or "physician*" or "clinician*" or "hospital medical staff" or "nurs* or doctor* or pharmacist* |
|  | Physician |  |  |  |
|  | Pharmacist |  |  |  |
|  | Nurse |  |  |  |
| Facet 3 | Inpatients | - Inpatient - Hospitalised patient - Hospital patient | | inpatient* or hospitali*ed patient* or hospital patient* |
| Facet 4 | Workflow | - Working practice - Workaround - Workflow | | work* practice* or workaround* or work flow* or workflow* |
| **Number of records identified: 539** | | | | |
| **Pubmed (accessed on 19/11/18)** | | | | |
| **MeSH term** | | | **Keyword** | |
| Facet 1 | Electronic prescribing | | Electronic prescribing or CPOE or Medical order entry system or Medication alert system or Computerised physician order entry or Computerized physician order entry or Computerised provider order entry or Computerized provider order entry or e-prescribing or e prescribing or eprescribing | |
|  | Medical order entry system | |  |  |
| Facet 2 | Health personnel | | healthcare professional or health care professional or healthcare personnel or health care personnel or health personnel or doctor or physician or clinician or hospital medical staff or pharmacist or nurse or hospital nursing staff or registered nurse | |
|  | Physician | |  |  |
|  | Pharmacist | |  |  |
|  | Nurse | |  |  |
|  | Hospital medical staff | |  |  |
| Facet 3 | Inpatient | | Inpatient or hospitalised patient or hospitalized patient or hospital patient | |
| Facet 4 | Workflow | | Workflow or work flow or working practice or workaround or practice pattern or communication or staff time | |
|  | Communication | |  |  |
| **Number of records identified: 483** | | | | |
| **Total number of records identified: 1476** | | | | |
