# Supplementary material for: The impact of electronic prescribing systems on healthcare professionals’ working practices in the hospital setting: a systematic review and narrative synthesis
Source: BMC Health Serv Res. 2019 Oct 22;19:742. doi: 10.1186/s12913-019-4554-7 (PMC6806498; doi:10.1186/s12913-019-4554-7)
Supplement: Supplementary file 2 — Additional file 2: Table S2. Data extraction table. [file 12913_2019_4554_MOESM2_ESM.docx]

| **(In alphabetical order): First author,**  **Country, Year, Reference** | **Study aim(s)** | **Study design** | **Setting (Hospital/ward/speciality)** | **Population (type of healthcare professional)** | **Sample size** | **Duration of study** | **Electronic system used (type/brand if stated)** | **Comparator** | **Outcome measure(s)/Main findings** |
| --- | --- | --- | --- | --- | --- | --- | --- | --- | --- |
| Alsweed,  Saudi Arabia, 2014^30^ | Explain the impact of computerised provider order entry (CPOE) implementation on nursing workflow, patient safety and medication errors | Cross-sectional survey  (Quantitative) | Public hospital, inpatient units | Nurses | 96 questionnaires returned (85.7% response rate) | April-May 2012 (4 weeks) | CPOE-  Not specified | n/a | CPOE training has an important impact on various facets of clinical work, on the whole participants were satisfied with their workflow after CPOE implementation, those who received good quality training perceived that CPOE use reduced medication errors and improved patient safety |
| Armada,  Spain,  2014^33^ | **1)** Evaluate the effects of a CPOE system by detecting prescription errors (frequency, type, severity) **2)** Impact of electronic prescribing (EP) on working conditions and satisfaction of personnel | Longitudinal prospective controlled before-after study  (Quantitative) | Tertiary care university centre in Madrid/Intensive care/Acute cardiac care | Doctors and nurses | 470 treatment orders, 27 physicians and 20 nursing staff completed questionnaire | Jun - Dec 2013 - 3 sampling stages of 21 consecutive days each | CPOE-  Farmatools Dominion; Global Dominion Access SA, Bilbao, Spain | Paper | Most participants rated accessibility to EP program as good to very good but also the overall performance and other 13 aspects regarding CPOE implementation. When asked about workload - 74% of physicians and 17% nurses considered this negative, physicians said it took more time to order and overdependence on technology |
| Ayatollahi,  Iran,  2015^34^ | Investigate physicians’ and nurses’ opinions about the impact of CPOE on their workflow | Cross-sectional survey  (Quantitative) | General hospital with 199 beds | Doctors and nurses | 101 nurses (69.7%), 3 specialist physicians (18.8%), 10 general practitioners (83.8%) | Not specified | CPOE-  Not specified | n/a | Positive impact CPOE: patient safety, inter-organisational workflow, working relationship between physicians and nurses, quality of patient care, nurses were more satisfied with the positive impact of CPOE on their relationship with physicians |
| Barber,  UK,  2007^10^ | Formative socio-technical evaluation of a pilot implementation of an integrated electronic prescribing, automated dispensing, barcode patient identification and electronic medication administration record (EMAR) system on one ward | Qualitative observational approach | Surgical ward in a teaching hospital | Doctors, nurses, pharmacists and hospital managers | Interviews with 9 nurses, 5 doctors, 3 pharmacists, 1 hospital manager), 1 focus group consisting of 5 doctors, 5 nurses, 3 pharmacists, 1 hospital manager | Focus group held 9 months after implementation | EPMA (electronic prescribing and medication administration system) -  ServeRx | n/a | Summary of findings mapped onto a structured evaluation framework: System function, human perspective and organisational context. Attitudes to the system in the early stages were mixed. Over time, and with experience of making the system work for them, staff attitudes changed to become more balanced and the potential benefits of the system became clearer to most. |
| Baysari,  Australia, 2018^28^ | Explore the views of nurses and doctors during the very early stage of implementation of a CPOE system in a pediatric hospital, and then to examine changes in perceptions and reported behaviors over the course of the shakedown period, as use of the CPOE system became routine. | Longitudinal qualitative study | Acute paediatric tertiary hospital, medical and surgical wards | Nurses and doctors | 122 (86 nurses, 36 doctors) | 6 months | CPOE-  Cerner Millennium Powerchart | Paper | Unfamiliarity with the system was perceived as a key attribute to influencing both the time it took to complete tasks and medication safety. CPOE systems had resulted in stress and disuse of the system. During early interviews it was perceived that there was a reduction in patient interaction but the emphasis on reduced patient interaction declined in later interviews. Prescribing and medication administration took longer due to additional steps compared to paper. Due to an increase in time to complete tasks, workarounds were introduced. |
| Bedouch,  France, 2012^25^ | (1) what are the characteristics of PIs in terms of the drugs involved, DRP description, pharmacists’ recommendations and the physicians’ acceptance or not? (2) What method(s) do pharmacists use to communicate their PIs when a CPOE system is available? (3) What are the independent predictors of acceptance of the recommendation by the physician? | Prospective cohort study  (Quantitative) | Teaching hospital (Grenoble University Hospital), seven medical wards: cardiology (27 beds), geriatrics (40 beds), infectious disease (27 beds), internal medicine (22 beds), nephrology (21 beds), respiratory medicine (18 beds) and rheumatology (27 beds) | Pharmacists | 448 pharmacist interventions | 10 weeks | CPOE-  Cristalnet, CRIH des Alpes, Grenoble, France (home-grown system) | n/a | Physicians accepted 86.6% of pharmacists interventions, time for acceptance was less than 1 hour in 50% of cases, pharmacists preferred face-to-face communications |
| Beuscart-Z'ephir,  France,  2005^26^ | Analyse the impact of medication ordering and administration functions of CPOE on doctor—nurse communications and cooperation | Observational and cognitive psychology and ergonomics  (mixed methods) | 3 French hospitals: 1) 413 bed public hospital, 2) 3000 bed university hospital 3) 825 bed university hospital | Physicians and nurses | site 1: 450 hours of observations, site 2: 80 hours of observations, site 3: 60 hours of observation  Number of interviews not specified | Not specified | All sites - CPOE  site 1: DxCare, site 2: paper with early CPOE, site 3: complete PICS including MEDASYS DxCare component | paper at site 2 | Physicians and nurses cooperation and coordination impacted by CPOE. Physicians and head-nurses staff should be particularly concerned with the necessity of maintaining or imposing time slots dedicated to physician—nurse synchronous oral communications. |
| Burgin,  UK,  2014^11^ | To establish the changes that electronic systems afforded to pharmacist’s work practices, and to understand how and why pharmacists in a large UK teaching hospital had responded to these changes. | Qualitative – focus groups | Large acute NHS teaching hospital | Pharmacists | 20 pharmacists (4-6 pharmacists per focus group) | The focus groups were conducted at 1–2 weekly intervals | EPMA-  Not specified | Paper | Pharmacists highlighted three main overarching themes: reduced patient contact, documentation in electronic patient records and professional representation in the clinical environment had all been impacted by the introduction of an electronic medical record and electronic prescribing and medication administration system |
| Davies,  UK,  2017^12^ | Assess the impact of EP system on safety culture | Cross-sectional survey  (Quantitative) | Dorset County Hospital - Surgical patients | Clinicians, nurses and pharmacists | 82/238 responses (34.5%) | 2 weeks (6 weeks after EP implementation) | EP-  JAC Medicines management | n/a | Clinicians had more negative responses than positive about the EP system, ease of prescribing but safety and time concerns |
| Franklin,  UK,  2007^13^ | Assess the impact of closed-loop electronic prescribing and medication administration (EPMA), automated dispensing and barcode scanning on prescribing and administration errors, confirmation of patient identity and staff time | Uncontrolled before and after design  (Quantitative) | Teaching hospital, 28 bed general surgery ward | Doctors, nurses and pharmacists | Ward pharmacist self-reported the time taken to provide a clinical service to the study ward each day for 4 weeks, time taken to carry out each scheduled non-IV drug round was recorded for nurses | 3-6 months before and 6-12 months after the intervention | EPMA-  ServeRx | paper | Pharmacist took an extra 24 seconds post implementation of EPMA, drug rounds were quicker for nurses but a higher percentage of time was spent on medication related tasks between drug rounds |
| Holden,  USA,  2010^17^ | To identify and describe physicians beliefs about the use of electronic medical records and CPOE for inpatient and outpatient care to build an understanding of what factors shape information technology use behaviour in the unique context of health care delivery | Semi-structured qualitative research | Two large Midwest US hospitals | Physicians | 20 Physicians | Hospital 2 - 7 months of order entry, information re. hospital 1 not specified | CPOE-  Commercial EMR (electronic medical records) and system (not specified) | n/a | Use improved the ease of personal performance, information easier to access, having all the information was thought to improve clinical decision making, over-reliance on technology, perceived to improve communication between colleagues and nurses |
| Hollister,  USA,  2011^18^ | Describes a project designed to increase computerized physician order entry in a community hospital staffed by voluntary and employed physicians | Uncontrolled before and after study  (Quantitative) | Greenwich hospital | Physicians | Not specified | December 2008 - May 2009 (unclear when implementation took place) | CPOE-  Meditech (Canton Massachusetts) during time of the study was running on the Magic 5.63 version | paper | Time taken for medication orders to be verified by pharmacy reduced. CPOE reduced the total time from medication ordering to patient delivery and thus contributed to improved patient care |
| Khajouei,  Netherlands, 2011^21^ | To study the satisfaction of end-users of a computerized physician order entry (CPOE) system concerning ease of use and the effect on users’ workflow, efficiency, and medication safety and to seek users’ opinions regarding required improvements of the system. | Cross-sectional survey  (Quantitative) | Academic medical centre, 1002 bed university hospital, Amsterdam | Physicians and nurses | 106/217 physicians and 327/587 nurses responded | Not specified | CPOE-  Medicator | n/a | High satisfaction concerning the effect of medicator on their workflow, physicians emphasised that the system facilitated the coordination of activities with nurses, pharmacists and other physicians |
| Mehta,  UK,  2009^14^ | Describe how EP has changed the way pharmacy staff in UK hospitals work, establish the perceived advantages and disadvantages, establish the benefits to pharmacy department of changing from a manual system to an electronic system of prescribing | Qualitative study - Interview based via a semi structured interview | 7 hospitals | Pharmacists | 7 interviews | March - April 2005 | EP-  3 hospitals used Meditech system, 2 used the TDS 7000 system and 2 used JAC system | Paper | More prescriptions screened by pharmacists, more time available for pharmacists to have more clinical input on wards and attending more ward rounds, at 3 hospitals pharmacy staff were able to carry out more clinical activities without increasing the amount of time spent at ward level, when short staffed some pharmacist would review medication from dispensary, reduced contact time with patients, 6 hospitals said they had a quicker turnaround time for discharge prescriptions and inpatient items when sent electronically, 3 hospitals said the pharmacy workload increased |
| Mekhjian,  USA,  2002^19^ | To evaluate the benefits of CPOE and electronic medication record on the delivery of health care | Uncontrolled before and after design (time and motion study - Quantitative) | Ohio State university health system - academic medical centre that comprises of 4 hospitals | Physicians | 46 medication events before CPOE and 70 medication events after CPOE were observed | Pre-EPMA - Jan - Feb 2000, Post-EPMA - May - Jun 2000 | CPOE-  Invision 24 with graphical user interface (Siemens medical solutions health services Corp) | Paper | Medication turnaround time decreased from 5hr 28 mins to 1hr 51 mins (64% reduction) - two key phases that was improvement were communication of the order to pharmacy and administration of the dispensed medication to the patient |
| Niazkhani,  Netherlands, 2009^24^ | To compare the perceived impact of CPOE system on nursing medication practice - questionnaire administered before and after implementation of CPOE | Uncontrolled before and after survey study  (Quantitative) | Erasmus University medical centre, 1237 bed academic hospital | Nurses | 154/295 nurses (52.2%) pre-implementation and 136/304 nurses (44.7%) post-implementation | Questionnaires sent 2 weeks prior to implementation and sent 5 months after implementation | CPOE-  Medicatie/EVS | 2 paper based systems (Kardex systema nd TIMED system) | When nurses were asked if they would want to change their current process back to paper they responded that they would prefer to continue on a CPOE system but the Mann-Whitney U test showed nurses believed the CPOE system did not support their work processes more than the paper-based system, 56.7% of respondents commented that the post-CPOE workflow had become less efficient, although they were generally satisfied with the system |
| Niazkhani,  Netherlands, 2010^23^ | To assess the effects of a CPOE system on inter-professional workflow in the medication process | Qualitative - semi structured interviews | Erasmus University medical centre, 1237 bed academic hospital | Physicians, pharmacists, nurses and a pharmacy technician | 23 semi-structured interviews (12 nurses, 8 physicians, 2 pharmacists and 1 pharmacy technician) | November 2006 - June 2007 | CPOE-  Medicatie/EVS, iSoft Leiden, the Netherlands | n/a | System benefitted physician-pharmacy and nurse-pharmacy while impeding the physician-nurse workflow |
| Niazkhani,  Netherlands, 2011^22^ | To evaluate the problems experienced after implementing a CPOE system, their possible root causes and the responses of providers in order to incorporate the system into their daily workflow | Qualitative study - semi structured interviews, artefacts from their daily work, educational material to train physicians and nurses to use the CPOE system | Erasmus University medical centre, 1237 bed academic hospital | Physician, nurses and pharmacists | 21 semi-structured interviews with clinicians, 6 physicians and 12 nurses from adult inpatients, 2 pharmacists, 1 senior pharmacy technician | late 2006 - early 2007 | CPOE-  Medicatie/EVS (version 2.3) and iSoft (now iSofthealth) |  | Findings based on the 5 stages of medication-use cycle, **Prescribing**: CPOE not accessible by patient bed therefore physicians usually rely on their memory of the list when visiting the patient, doctors make the changes to patient medication and add medicines after the round is complete, as it can take a few hours for orders to be written electronically nurses request the physician to write some medicines on paper as a temporary prescription, **communication**: nurses miss the stickers printed with new prescriptions if not communicated by a physician face-to-face or over the phone, **dispensing**: high medicines returns rate added to pharmacy workload initially but now the nurses have to select the non-stock items they want from the pharmacy, **administration:** nurses usually started administering the medication before they received the printed out label, hand changing the labels but no record on CPOE/information not communicated to physician, **monitoring:** issues in the prescribing phase may partly overlap with those in the monitoring phase |
| Pelayo,  France,  2013^27^ | To compare the impact of CPOE implementation and of the workplace organizational determinants on the doctor–nurse cooperation and communication processes. | Naturalistic observations supported by handwritten field notes and interviews of those who were observed  (Qualitative) | 3 hospitals sites - 1 - Academic hospital (825 beds + CPOE system, medication orders functions in use for 6 months), 2 - not academic hospital (618 beds + CPOE system in use for 4 years), 3 - Academic hospital (3500 beds + paper based system) | Doctors and nurses | Study 1 - 60 observations and 49 interviews, study 2 - 194 hours of observations (23 doctors and 25 nurses) | Not specified | CPOE -  Hospital 1 and 2 - commercial systems, Hospital 3 – paper based | Paper | Technical system has no significant impact on the cooperative activities within the organisation. CPOE does not cause a different in the dialogues' duration or contents and does not seem to deteriorate the doctor-nurse communications |
| Pontefract, UK, 2018^15^ | The aim of this  study is to explore pharmacists and physicians perceptions of their interprofessional communication  in the context of the technology and whether electronic messaging and CDS has an impact on this. | Qualitative – focus groups and interviews | Two acute hospitals: the University Hospitals Birmingham  NHS Foundation Trust (UHBFT) and Guy’s and St Thomas’ NHS Foundation Trust (GSTH) | Pharmacists and physicians | Four focus groups were conducted between 2014 and 2015; two uni-professional focus groups and one mixed focus group were conducted at UHBFT, and one mixed at GSTH. | Focus groups conducted between 2014-2015 | CPOE-  Locally developed system – PICS (UHBFT) and commercial CareVue (Critical Care) MedChart (In-patient  wards) | Paper | Three predominant themes; increased communication load; impaired decision-making; and improved workflow. New technical role introduced for the pharmacist and stated they were unable to ‘fine tune’ prescriptions as they previously could on paper. Technology has removed their power to make ‘low risk’ amendments. The technology was found to increase the frequency with which the pharmacist needed to intervene with the physician. Face to face communicate was preferred. |
| Saddik,  Saudi Arabia,  2014^31^ | To explore nurses’ perceptions regarding the CPOE and its impact on nurse-physician communication in the medication order process. | Cross-sectional survey  (Quantitative) | 112 bed hospital | Nurses | 174 nurses invited to complete questionnaire - 146 responded (83%) | Not specified | CPOE-  Not specified | n/a | Almost all of the nurses perceived that CPOE allowed easier accessibility to patients’ medication records and provided complete and legible drug prescriptions. The majority of nurses agreed that more physician contact was required with CPOE and that the physician was always followed up by phone call regarding certain prescriptions. Almost all of the nurses perceived that CPOE supported their work process. |
| Van Wilder,  UK,  2016^16^ | To explore how EPMA may affect different aspects of nurses' work, relating to both workload and patient safety | Observational - uncontrolled before and after study  (Quantitative) | 14 bed medicine for the elderly ward/London teaching hospital | Nurses | 20 drug rounds pre-EPMA (22hrs) and 14 drug rounds post- EPMA (18 hrs), 9 different nurses observed pre-EPMA and 11 nurses observed post-EPMA | One month before implementation and continuing until one month after (Feb 2015 - Apr 2015) | EPMA-  Commercial system (not specified) | Paper | Overall findings presented in to the work conducted in one study based in the UK suggested that the introduction of an EPMA system did not significantly affect the length of time spent on a drug administration round but altered the distribution of tasks with a doubling of the time spent on documentation, zero time spent looking for drug charts post EPMA, documentation time doubled post-EPMA |
| Weir,  USA,  1996^20^ | The purpose of this study was to examine nurses perceptions of the impact of POE on three general dimensions - quality of care, communication patterns between physicans and nurses and combined perceptions of control, perceptions of personal competence and the interest in the job | Cross sectional survey  (Quantitative) | 8 hospitals (4 had POE implemented and 4 used clerking entry of orders) | Full time registered nurses who had worked on that ward for at least 1 year | 201 out of 605 surveys returned (33% response rate) | Not specified | CPOE-  OE/RR 2.5 | Comparing hospitals that have the same computer system but differ in terms of CPOE implementation - allows for a more precise evaluation of the impact of POE itself | Nurses working in POE environments perceived their computer system as having more of a positive benefit to patient care than nurses working with a similar computer system where POE had not been implemented, computer system made them feel more competent at their job, fewer errors, more time with patients, documentation was complete and overall relationship with physicians were improved, some perceptions of decreased control, nurses working in a POE environment reported no difference in their perceived access to physicians than nurses working in a non-POE environment - suggests that computers did not decrease the need to talk to physicians |
| Wenzer,  Denmark, 2006^32^ | how medication is enacted at two Danish, internal medical wards | A socio-technical study - observations, interviews and analysis of the user interface and of other documents  (Qualitative) | 2 internal medical ward in a hospital | Physicians and nurses | 48 hours of observations, 6 interviews (2 physicians and 4 nurses) | Not specified | CPOE-  Commercial system developed by Systematic, Aarhus, DK | n/a | Login procedures were time consuming - doctors would leave themselves signed in so nurses could make the changes, paper-copies were back up and used on average twice a week for hours as the system was unstable, information not clear for patients on discharge via the CPOE print out therefore nurses would write an additional medication guide to help the patient, higher cognitive pressure on physicians and nurses memory skills, system had no CDS, communication between physicians, nurses and patients was not supported but demanded considerably work-around |
| Westbrook,  Australia, 2013^29^ | To quantify and compare the time doctors and nurses spent on direct patient care, medication related tasks, and interactions before and after electronic medication management system (eMMS) introduction. | Controlled pre–post, time and motion study using the WOMBAT tool  (Quantitative) | 400 bed major public hospital | Doctors and nurses | **Baseline** - 30 nurses (3 wards), 133.71 hours, 59 doctors (4 wards), 150.88 hours, **Post** - 40 nurses (2 control and 1 intervention ward), 143.73 hours, 39 doctors (2 control and 2 intervention wards), 205.38 hours | Pre - July 2005 - march 2006 (nurses) and July 2006 - December 2006 (doctors), Post minimum 9 months after intervention for nurses and 14 months for doctors | CPOE-  Cerner Millennium Powerorders system | Paper | Implementation of the eMMS was not associated with significant changes in the proportions of time doctors and nurses spent on direct patient care or medication-related tasks, relative to their colleagues on the control wards. Task time redistribution did occur within some specific areas. |
