# Supplementary material for: The impact of electronic prescribing systems on healthcare professionals’ working practices in the hospital setting: a systematic review and narrative synthesis
Source: BMC Health Serv Res. 2019 Oct 22;19:742. doi: 10.1186/s12913-019-4554-7 (PMC6806498; doi:10.1186/s12913-019-4554-7)
Supplement: Supplementary file 3 — Additional file 3: Table S3. Quality assessment - The Mixed Methods Appraisal Tool (MMAT) version 2018. [file 12913_2019_4554_MOESM3_ESM.docx]

|  | **1. Qualitative** | | | | | **3. Quantitative non-randomised** | | | | | **4. Quantitative descriptive** | | | | | **5. Mixed methods** | | | | | **Score* (%)** |
| --- | --- | --- | --- | --- | --- | --- | --- | --- | --- | --- | --- | --- | --- | --- | --- | --- | --- | --- | --- | --- | --- |
| **(In alphabetical order): First author, Country, Year, Reference** | **1.1** | **1.2** | **1.3** | **1.4** | **1.5** | **3.1** | **3.2** | **3.3** | **3.4** | **3.5** | **4.1** | **4.2** | **4.3** | **4.4** | **4.5** | **5.1** | **5.2** | **5.3** | **5.4** | **5.5** |  |
| Alsweed,2014^30^ |  |  |  |  |  |  |  |  |  |  | Yes | Yes | Yes | Yes | Yes |  |  |  |  |  | 100 |
| Armada, 2014^33^ |  |  |  |  |  | No | Yes | Can't tell | Can't tell | Yes |  |  |  |  |  |  |  |  |  |  | 50 |
| Ayatollahi, 2015^34^ |  |  |  |  |  |  |  |  |  |  | Yes | Yes | Yes | Yes | Yes |  |  |  |  |  | 100 |
| Barber, 2007^10^ | Yes | Yes | Yes | Yes | Yes |  |  |  |  |  |  |  |  |  |  |  |  |  |  |  | 10 |
| Baysari, 2018^28^ | No | Yes | Yes | No | Yes |  |  |  |  |  |  |  |  |  |  |  |  |  |  |  | 50 |
| Bedouch, 2012^25^ |  |  |  |  |  | Yes | Yes | Can't tell | Can't tell | Yes |  |  |  |  |  |  |  |  |  |  | 50 |
| Beuscart-Z'ephir, 2005^26^ | Yes | Can't tell | Can't tell | Can't tell | No |  |  |  |  |  | Can't tell | Can't tell | Yes | Can't tell | Can't tell | Yes | Yes | No | No | Can't tell | 25 |
| Burgin, 2014^11^ | Yes | Yes | Yes | Yes | Yes |  |  |  |  |  |  |  |  |  |  |  |  |  |  |  | 100 |
| Davies, 2017^12^ |  |  |  |  |  |  |  |  |  |  | Yes | Yes | Yes | No | No |  |  |  |  |  | 50 |
| Franklin, 2007^13^ |  |  |  |  |  | Yes | Yes | Yes | Can't tell | Yes |  |  |  |  |  |  |  |  |  |  | 75 |
| Holden, 2010^17^ | Yes | Yes | Yes | Can't tell | Yes |  |  |  |  |  |  |  |  |  |  |  |  |  |  |  | 75 |
| Hollister, 2011^18^ |  |  |  |  |  | Can't tell | Yes | Yes | Can't tell | Yes |  |  |  |  |  |  |  |  |  |  | 50 |
| Khajouei, 2011^21^ |  |  |  |  |  |  |  |  |  |  | Yes | No | Yes | Can't tell | Yes |  |  |  |  |  | 50 |
| Mehta, 2009^14^ | Yes | Yes | Yes | Yes | Yes |  |  |  |  |  |  |  |  |  |  |  |  |  |  |  | 100 |
| Mekhjian, 2002^19^ |  |  |  |  |  | Can't tell | Yes | Yes | Can't tell | Yes |  |  |  |  |  |  |  |  |  |  | 50 |
| Niazkhani, 2009^24^ |  |  |  |  |  | Yes | Yes | Yes | Can't tell | Yes |  |  |  |  |  |  |  |  |  |  | 75 |
| Niazkhani, 2010^23^ | Yes | Yes | Yes | Yes | Yes |  |  |  |  |  |  |  |  |  |  |  |  |  |  |  | 100 |
| Niazkhani, 2011^22^ | Yes | Yes | Yes | No | Yes |  |  |  |  |  |  |  |  |  |  |  |  |  |  |  | 75 |
| Pelayo, 2013^27^ | Yes | Yes | Yes | Yes | Yes |  |  |  |  |  |  |  |  |  |  |  |  |  |  |  | 100 |
| Pontefract, 2018^15^ | Yes | Yes | Yes | Yes | Yes |  |  |  |  |  |  |  |  |  |  |  |  |  |  |  | 100 |
| Saddik, 2014^31^ |  |  |  |  |  |  |  |  |  |  | Yes | Yes | Yes | Yes | Yes |  |  |  |  |  | 100 |
| Van Wilder, 2016^16^ |  |  |  |  |  | Can't tell | Yes | Yes | Can't tell | Yes |  |  |  |  |  |  |  |  |  |  | 50 |
| Weir, 1996^20^ |  |  |  |  |  |  |  |  |  |  | No | Yes | Yes | No | Yes |  |  |  |  |  | 50 |
| Wenzer, 2006^32^ | Yes | Can't tell | Yes | Can't tell | No |  |  |  |  |  |  |  |  |  |  |  |  |  |  |  | 50 |
| Westbrook, 2013^29^ |  |  |  |  |  | Yes | Yes | Yes | Yes | Yes |  |  |  |  |  |  |  |  |  |  | 100 |

* meets % of the MMAT criteria
